# Study on Friction and Wear Properties of Mo_2_C-Coated Ultrahard TZM Alloy by High-Temperature Gas-Phase Carburization

**DOI:** 10.3390/ma19102022

**Published:** 2026-05-13

**Authors:** Shilei Li, Jing Liang, Li Yu, Weiwei Zhang, Tian Chang, Yu Xia, Kai Chen, Wen Zhang, Yanchao Li, Hailong Xu, Jianfeng Li

**Affiliations:** 1 Center for Advancing Materials Performance from the Nanoscale (CAMPNano), State Key Laboratory for Mechanical Behavior of Materials, Xi’an Jiaotong University, Xi’an 710049, China; 2 Northwest Institute for Non-Ferrous Metal Research, Xi’an 710016, China; 3 Xi’an Noble Metal Material Co., Ltd., Xi’an 710201, China

**Keywords:** TZM alloy, Mo_2_C-coating, hardness, friction and wear properties

## Abstract

In this study, a continuous coating with a thickness of 20 μm and intimate bonding to the substrate was in situ fabricated on the TZM alloy (Mo-0.6Ti-0.08Zr-0.04C) via high-temperature gas-phase carburization at 1200 °C combined with water quenching, using CO as the carbon transport carrier. The coating possesses a fine equiaxed grain structure with an average grain size of 1.48 μm, and its microhardness reaches 1479 ± 42 HV. This modification process does not sacrifice the inherent strength and ductility of the TZM alloy matrix, while it does reduce the wear volume of the alloy by 78.8% in comparison with the uncoated rolled TZM alloy.

## 1. Introduction

Titanium–zirconium–molybdenum (TZM) alloy, as a typical representative of molybdenum-based high-temperature structural materials, plays an irreplaceable role in high-end fields such as aeroengine nozzles, nuclear reactor components, and semiconductor manufacturing equipment [1,2,3]. However, the inherent drawbacks of TZM alloy, including low hardness (approximately 200–400 HV) [4,5] and insufficient wear resistance, severely restrict its service life under frictional conditions and limit its further application in scenarios such as die-casting molds and high-temperature wear-resistant components.

Primarily, dry contact is inevitable in most applications of molybdenum-based alloys, which leads to oxidation wear [6]. But Mo_4_O_11_, serving as a solid lubricant, would be generated from MoO_3_ due to friction heat and lattice shear during dry contact at room temperature, forming the mixed oxides of MoO_3_ and Mo_4_O_11_, which could strongly influence wear behaviors and mechanisms [7,8,9]. To address this issue, researchers have attempted to improve the wear resistance of the TZM alloy via surface coating techniques. Traditional MoSi_2_ oxidation-resistant coatings suffer from high room-temperature brittleness and low-temperature pesting and oxidation [10,11]. Furthermore, MoSi_2_ coatings fail to preserve the original strength and ductility of the substrate [12]. Therefore, developing a coating system that can significantly enhance surface hardness and wear resistance without impairing the strength and ductility of the TZM alloy substrate has become a critical requirement for expanding its engineering applications.

Molybdenum carbides (Mo_2_C, MoC), as typical transition metal carbides, possess high hardness (1800–2200 HV), excellent wear resistance, and good thermodynamic compatibility with molybdenum-based materials [13,14]. Their hexagonal close-packed (HCP) crystal structure endows the coatings with favorable thermal and mechanical stability, making them ideal candidates for wear-resistant coating constituents. Accordingly, in this study, molybdenum carbide coatings were fabricated on TZM alloy for surface modification through high-temperature gas-phase carburization. On the premise of retaining the outstanding strength and ductility of the TZM alloy, the surface hardness and wear resistance were greatly improved.

Conventional MoSi_2_ oxidation-resistant coatings suffer from inherent drawbacks such as room-temperature brittleness, low-temperature pest degradation, and poor wear resistance [15,16]. Deposited coatings prepared by PVD, CVD and other techniques exhibit weak interfacial adhesion and a large heat-affected zone, failing to achieve the synergistic retention of coating strengthening and matrix strength–ductility [17]. Targeting the research gap regarding the strength–toughness imbalance in the surface modification of TZM alloys, this study originally proposes non-contact high-temperature vapor carburization coupled with water quenching technology to in situ fabricate high-purity and ultrafine Mo_2_C coatings. The developed coating remarkably enhances the hardness and wear resistance while completely maintaining the strength and ductility of the TZM substrate. Meanwhile, the gaseous cyclic carbon supply mechanism of CO is revealed. This work provides a novel insight for the design and engineering application of high-performance molybdenum-based high-temperature wear-resistant materials.

## 2. Materials and Experiment

TZM alloy (Mo-0.6Ti-0.08Zr-0.04C) sheets were prepared by powder metallurgy combined with rolling processing. A small crucible was placed inside a large crucible, and graphite powder was filled between the two crucibles. The TZM alloy sheets were put into the small crucible filled with argon. The two covered crucibles were heated in a muffle furnace to 1200 °C and held for 12 h, followed by quenching in water. The Vickers hardness of the alloy was measured using a 401MVD semi-automatic micro Vickers hardness tester (Wolpert Measuring Instruments (Shanghai) Co., Ltd, Shanghai, China) with a load of 500 g and a dwell time of 15 s. Thirty indentations were made on each sample, and the average value was calculated. The friction and wear properties at room temperature in air were tested on a GHT-1000 friction and wear tester (Lanzhou Zhongke Kaihua Technology Development Co., Ltd., Lanzhou, China), with a 316L stainless-steel ball as the friction pair, under a normal load of 15 N, a sliding amplitude 1 mm, a frequency of 5 Hz, and a testing time of 20 min, with three replicates performed for each sample. The wear morphology and wear volume of the samples were characterized using an optical microscope equipped with a 3D data micro-operating system (VHX-7000N, KEYENCE, Osaka, Japan). The coating morphology and elemental distribution were characterized by a Phenom Prox desktop scanning electron microscope (SEM) (Thermo Fisher Scientific, Eindhoven, The Netherlands). The grain size of the samples was measured by the intercept method. The phase composition of the samples was analyzed using a D8 ADVANCE high-resolution X-ray diffractometer (XRD) (Bruker AXS, Karlsruhe, Germany). Tensile tests were conducted in air at room temperature using a WAW-600B/E electrohydraulic servo testing machine (Jinan Liangong Testing Technology Co., Ltd., Jinan, China) (strain rate 0.5 mm/s), with three replicates performed for each sample.

## 3. Results and Discussions

### 3.1. Coating Morphology and Elemental Composition

**Figure 1:**
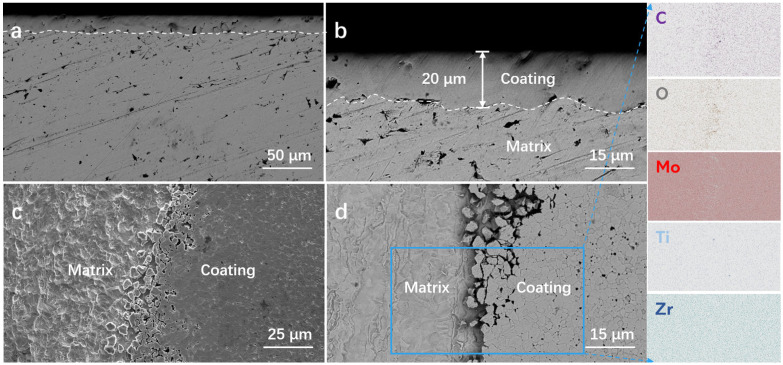
Microstructure and EDS elemental distribution of the coating on TZM alloy fabricated via high-temperature gas-phase carburization and quenching treatment. (**a**,**b**) Cross-sectional morphologies and (**c**,**d**) surface morphologies.

Figure 1 presents the micromorphology and EDS elemental distribution of the coating on TZM alloy after high-temperature gas-phase carburization and quenching treatment. As shown in Figure 1a,b, a continuous and uniform reinforced coating is formed on the surface of the carburized sample. The coating exhibits a clear interface and tight bonding with the matrix, with no obvious cracks or spallation observed, and the average thickness of the coating is approximately 20 μm. After artificial peeling (Figure 1c,d), the coating microstructure exhibits fine equiaxed grain characteristics. Statistical analysis via ImageJ 1.47T software reveals that the average grain size of the coating is approximately 1.48 μm. EDS mapping results demonstrate that the C element is significantly enriched in the coating region, while the O element content is lower than that in the matrix.

**Figure 2:**
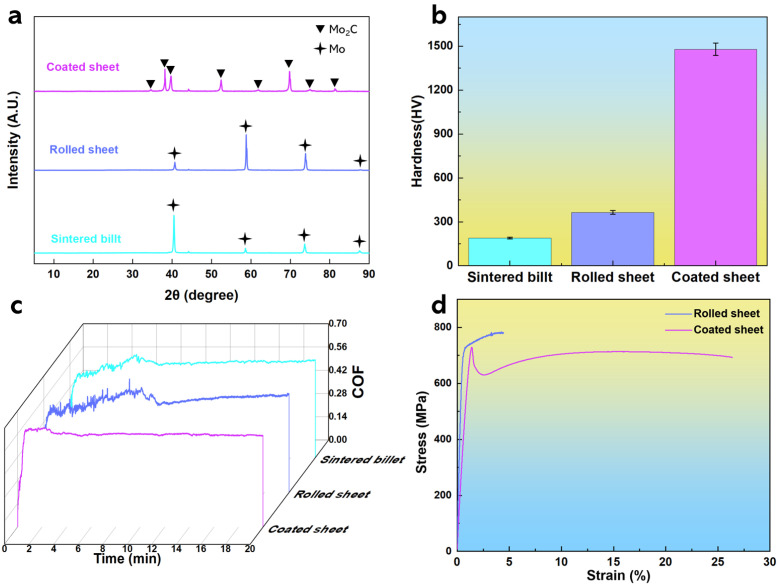
(**a**) XRD patterns of TZM alloy, (**b**) Vickers hardness of TZM alloy, (**c**) room-temperature coefficient of friction (CoF) of TZM alloy, and (**d**) room-temperature stress–strain curves of TZM alloy.

The XRD diffraction pattern is shown in Figure 2a. Only the characteristic diffraction peaks of the Mo matrix are present in the XRD spectrum of the sintered TZM alloy. The peaks at 2θ = 40.5°, 58.6°, 73.7°, and 87.7° correspond to the (110), (200), (211), and (220) crystal planes of Mo, respectively. The volume fractions of other elements are below the phase detection limit of XRD (typically 3–5%), so they cannot be effectively identified. No new phases are formed in the XRD spectrum of the rolled TZM sheet. However, the intensity of the diffraction peak corresponding to the (200) crystal plane increases sharply, while the intensity of the originally dominant (110) peak decreases substantially. This is a direct result of grain texture evolution during the rolling deformation. All characteristic diffraction peaks of the rolled TZM sheet shift slightly toward higher diffraction angles, which is caused by the lattice contraction of the Mo matrix induced by plastic rolling deformation.

For the coated TZM sample, strong characteristic diffraction peaks corresponding to the Mo_2_C phase are observed. This indicates that during the surface modification process, the Mo matrix reacts in situ with the carbon source, forming a continuous Mo_2_C ceramic modified layer on the surface of the TZM sheet.

### 3.2. Mechanical Properties and Friction-Wear Performance

As shown in Figure 2b, the hardness of the coated sample reaches 1479 ± 42 HV, which is increased by a factor of 4.1 compared with the rolled TZM sheet and 7.8 compared with the sintered TZM billet, and is also significantly higher than the hardness of TZM alloys reported in the previous literature [18,19,20]. The coefficients of friction (CoFs) of the sintered TZM billet, rolled TZM sheet and coated sample are 0.56, 0.56 and 0.55, respectively, which are similar to those reported for TZM alloys [19,21]. The CoF of the coated sample fluctuates briefly for 2.8 min and then tends to be stable, whereas those of the sintered and rolled samples exhibit gradual stabilization after 7.2 min and 9.8 min, respectively. In addition, the CoF of the rolled TZM sheet shows a continuous upward trend (as shown in Figure 2c). Room-temperature tensile results demonstrate that the TZM alloy modified by high-temperature gas-phase carburization and quenching maintains consistent strength and ductility with the solid solution-quenched TZM sample (the strength–ductility mechanism of solution-quenched TZM alloy has been reported in our previous studies) [22,23], possessing a tensile strength of 730 MPa and an elongation of 26.4%, as shown in Figure 2d.

### 3.3. Friction and Wear Mechanism

**Figure 3:**
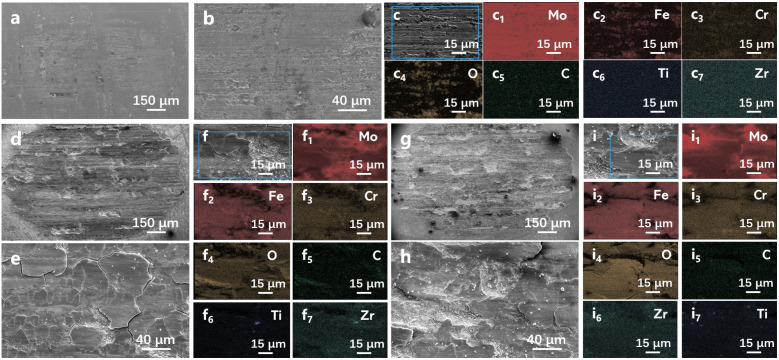
Macro- and micromorphologies of TZM alloys after friction and wear. (**a**–**c**) Coated sample, (**d**–**f**) sintered billet, (**g**–**i**) rolled sheet. (**c1**–**c7**), (**f1**–**f7**) and (**i1**–**i7**) are EDS mapping spectra of the blue rectangular regions in (**c**), (**f**) and (**i**), respectively.

Figure 3 shows the macro/micromorphologies of the coated sample, sintered billet and rolled TZM alloy after friction and wear, as well as the EDS elemental mapping of the corresponding regions. As can be seen from Figure 3a–c, the wear track of the coated TZM sample exhibits a narrow and shallow groove morphology, without obvious spallation, plastic flow or crack propagation. The extent of wear is significantly lower than that of the reported TZM alloys [4,11,18,19,20,21]. Only slight traces of surface plastic deformation are observed, indicating that the coating effectively resists the external load and frictional heat during the friction process. The EDS mapping reveals a uniform distribution of C, confirming that the integrity of the Mo_2_C-based composite coating is preserved during friction. The enrichment of Fe and Cr in the wear track indicates that the stainless-steel friction counterpart is detached and adheres to the coating surface during sliding. O is mainly concentrated in the wear track, demonstrating that the coating effectively suppresses high-temperature oxidation during the friction process.

As shown in Figure 3d–i, the friction regions of the sintered TZM billet and rolled sheet exhibit large-area material spallation. The wear tracks present a deep intertwined morphology of grooves and cracks with irregular wear craters, reflecting that severe brittle fracture and material detachment occur in the samples during the friction process. EDS mapping reveals a significant enrichment of Fe and Cr, indicating a severe material transfer from the 316 L stainless-steel friction counterpart. The soft Fe-based transferred layer forms abrasive particles at the friction interface, which aggravates the abrasive wear of the matrix. The O content rises substantially, while C exhibits a sparse distribution and relatively low concentration. Such morphological evolution is most likely attributed to the increase in the effective penetration depth of frictional heat, which accelerates the delamination of the oxide layer from the substrate [19]. In the stable wear stage, wear debris is expelled from the contact interface. The brittle oxides such as MoO_3_ formed by oxidation act as wear sources. According to the classical wear progression model, this marks the transition of TZM alloy from steady-state wear to an accelerated degradation state during the wear process [24]. The wear mechanism of both the sintered billet and rolled sheet is a composite mode consisting of oxidative wear, abrasive wear and brittle spallation [18,20].

**Figure 4:**
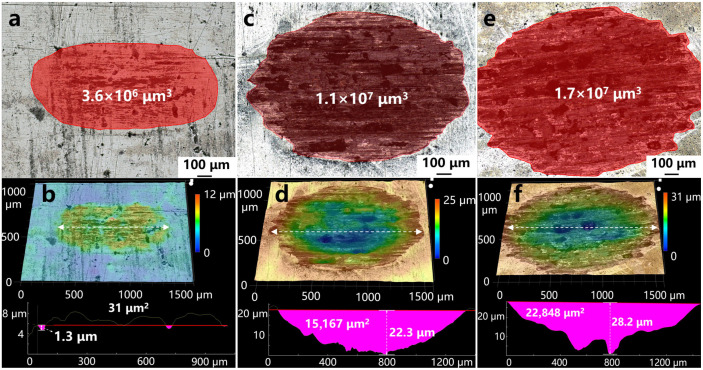
Quantitative wear volume, 3D depth-of-field composite morphology and wear track cross-sectional profiles of TZM alloys. (**a**,**b**) Coated sample, (**c**,**d**) sintered billet, (**e**,**f**) rolled sheet.

Figure 4 presents the quantitative wear volume, 3D depth-of-field composite morphology, and wear track cross-sectional profiles of the TZM alloys. As shown in Figure 4a,b, the total wear volume of the coated sample is merely 3.6 × 10^6^ μm^3^, accounting for 32.7% of that of the sintered billet and 21.2% of that of the rolled sheet, respectively. The 3D depth-of-field composite morphology and cross-sectional profiles further indicate that no obvious macroscopic depression exists in the wear track region, with a maximum wear depth of only 1.3 μm. The cross-sectional profile is nearly flush with the original surface, showing only extremely slight traces of material removal. The Mo_2_C-based composite coating greatly improves the resistance of the alloy surface to plastic deformation and abrasive cutting. It also effectively suppresses the material transfer from the friction counterpart and mitigates high-temperature oxidative wear. Only slight abrasive wear occurs in the coating during the friction process, without obvious material spallation and fatigue damage, which ultimately leads to a significant reduction in wear volume.

As shown in Figure 4c–f, the total wear volumes of the sintered TZM billet and sheet are 1.1 × 10^7^ μm^3^ and 1.7 × 10^7^ μm^3^, with maximum wear depths reaching 22.3 μm and 28.2 μm, respectively. The cross-sectional profiles show a wide and deep U-shaped feature, corresponding to the large-area brittle spallation and through-cracks observed in Figure 3d–i.

The room-temperature test results of this study can effectively predict the high-temperature service performance of TZM alloy. The as-prepared Mo_2_C coating features a single-phase structure, metallurgical bonding interface and high room-temperature hardness, which guarantee superior structural stability, wear resistance and deformation resistance at high temperatures. The alloy substrate maintains intact strength and ductility at room temperature, ensuring excellent high-temperature mechanical load-bearing capacity and thermal shock resistance. Moreover, the oxidation and wear resistance mechanisms of the coating at room temperature can be well extended to high-temperature working conditions, providing reliable support for the high-temperature engineering applications of the modified TZM alloy.

### 3.4. In Situ Formation Mechanism of Mo_2_C Coating

The spontaneity of carbide formation reactions is determined by Gibbs free energy calculations [5]. According to data from the Thermodynamic Handbook of Metals and Alloys, the standard Gibbs free energy (ΔG) of the reactions at 1200 °C (1473 K) satisfies ΔG < 0, verifying that reactions (1) and (2) can proceed spontaneously. However, MoC is a metastable phase, and the Gibbs free energy for the formation of Mo_2_C is lower, so Mo_2_C is the main phase in the coating. Although Ti and Zr can react with C to form carbides at high temperatures (reactions 3, 4), most Ti and Zr in the TZM alloy have already formed secondary phases during sintering. Only a negligible amount of Ti and Zr can further combine with C to form carbides in the subsequent carburization process, which is not discussed in detail here.


(1) $$2\mathrm{Mo}\left(s\right)+C\left(g\right)=Mo_{2}C\left(s\right)\;∆G_{1473\;K}=-130.2\;\mathrm{KJ}\cdot {\mathrm{mol}}^{-1}$$

(2) $$\mathrm{Mo}\left(s\right)+C\left(g\right)=\mathrm{MoC}\left(s\right)\;∆G_{1473\;K}=-123.1\;\mathrm{KJ}\cdot {\mathrm{mol}}^{-1}$$

(3) $$\mathrm{Ti}\left(s\right)+C\left(s\right)=\mathrm{TiC}\left(s\right)\;∆G_{1473\;K\;}=-39.9\;\mathrm{KJ}\cdot {\mathrm{mol}}^{-1}$$

(4) $$\mathrm{Zr}\left(s\right)+C\left(s\right)=\mathrm{ZrC}\left(s\right)\;∆G_{1473\;K}=-43.5\;\mathrm{KJ}\cdot {\mathrm{mol}}^{-1}$$

In this study, the TZM alloy has no physical contact with graphite powder, and the muffle furnace is operated under a non-absolute vacuum atmosphere. Trace amounts of oxygen (O_2_) and water vapor (H_2_O) remaining in the furnace undergo continuous redox reactions with graphite powder at 1200 °C (reactions 5 and 6), producing CO as the gaseous carbon carrier. After diffusing to the TZM alloy surface, the CO gas undergoes a high-temperature gas-phase carburization reaction, releasing carbon atoms that combine with elements on the alloy surface to form carbides, while generating CO_2_ (reaction 7). The formed CO_2_ gas diffuses back to the graphite powder surface, is reduced by graphite to regenerate CO, and continuously supplies the carbon source for the in situ formation of the Mo_2_C coating (reaction 8).


(5) $$C\left(s\right)+\frac{1}{2}O_{2}\left(g\right)=\mathrm{CO}\left(g\right)\;∆G_{1473\;K\;}=-57.7\;\mathrm{KJ}\cdot {\mathrm{mol}}^{-1}$$

(6) $$C\left(s\right)+H_{2}O\left(g\right)=\mathrm{CO}\left(g\right)+H_{2}\;∆G_{1473\;K}=-18\;\mathrm{KJ}\cdot {\mathrm{mol}}^{-1}$$

(7) $$2\mathrm{Mo}\left(s\right)+2\mathrm{CO}\left(g\right)=Mo_{2}C\left(s\right)+{\mathrm{CO}}_{2}(g)\;∆G_{1473\;K}=-82.2\;\mathrm{KJ}\cdot {\mathrm{mol}}^{-1}$$

(8) $$CO_{2}\left(g\right)+C\left(s\right)=2\mathrm{CO}\left(g\right)\;∆G_{1473\;K}=-20.7\;\mathrm{KJ}\cdot {\mathrm{mol}}^{-1}$$

## 4. Conclusions

In this study, a coating was fabricated on the surface of TZM alloy through high-temperature (1200 °C) gas-phase carburization and quenching. The phase composition and microstructure of the coating, as well as its effects on the microhardness and room-temperature friction-wear properties of TZM alloy, were systematically investigated. The main conclusions are as follows:

1. The coating consists of Mo_2_C with a thickness of 20 μm, and exhibits excellent interfacial bonding with the TZM substrate.
2. The microhardness of the Mo_2_C coating reaches 1479 ± 42 HV, which is 4.1 times that of the rolled TZM sheet.
3. The Mo_2_C coating reduces the wear volume of TZM alloy by 78.8% while maintaining its original strength and ductility, thus achieving a remarkable improvement in friction and wear performance.
4. CO serves as the carbon carrier for the growth of the Mo_2_C coating via high-temperature vapor transport, and continuously supplies carbon for the in situ formation of the coating.

## Data Availability

The original contributions presented in this study are included in the article. Further inquiries can be directed to the corresponding authors.
